# Convergence of IL-1β and VDR Activation Pathways in Human TLR2/1-Induced Antimicrobial Responses

**DOI:** 10.1371/journal.pone.0005810

**Published:** 2009-06-05

**Authors:** Philip T. Liu, Mirjam Schenk, Valencia P. Walker, Paul W. Dempsey, Melissa Kanchanapoomi, Matthew Wheelwright, Aria Vazirnia, Xiaoran Zhang, Andreas Steinmeyer, Ulrich Zügel, Bruce W. Hollis, Genhong Cheng, Robert L. Modlin

**Affiliations:** 1 Division of Dermatology, Department of Medicine, David Geffen School of Medicine at University of California Los Angeles, Los Angeles, California, United States of America; 2 Neonatal Research Center, Division of Neonatology and Developmental Biology, Department of Pediatrics, David Geffen School of Medicine at University of California Los Angeles, Los Angeles, California, United States of America; 3 Department of Microbiology, Immunology and Molecular Genetics, University of California Los Angeles, Los Angeles, California, United States of America; 4 School of Medicine, University of California San Francisco, San Francisco, California, United States of America; 5 Medicinal Chemistry, Bayer Schering Pharma AG, Berlin, Germany; 6 Common Mechanism Research Early Projects, Bayer Schering Pharma AG, Berlin, Germany; 7 Departments of Pediatrics, Biochemistry, and Molecular Biology, Medical University of South Carolina, Charleston, South Carolina, United States of America; New York University School of Medicine, United States of America

## Abstract

Antimicrobial effector mechanisms are central to the function of the innate immune response in host defense against microbial pathogens. In humans, activation of Toll-like receptor 2/1 (TLR2/1) on monocytes induces a vitamin D dependent antimicrobial activity against intracellular mycobacteria. Here, we report that TLR activation of monocytes triggers induction of the defensin beta 4 gene (DEFB4), requiring convergence of the IL-1β and vitamin D receptor (VDR) pathways. TLR2/1 activation triggered IL-1β activity, involving the upregulation of both IL-1β and IL-1 receptor, and downregulation of the IL-1 receptor antagonist. TLR2/1L induction of IL-1β was required for upregulation of DEFB4, but not cathelicidin, whereas VDR activation was required for expression of both antimicrobial genes. The differential requirements for induction of DEFB4 and cathelicidin were reflected by differences in their respective promoter regions; the DEFB4 promoter had one vitamin D response element (VDRE) and two NF-κB sites, whereas the cathelicidin promoter had three VDREs and no NF-κB sites. Transfection of NF-κB into primary monocytes synergized with 1,25D3 in the induction of DEFB4 expression. Knockdown of either DEFB4 or cathelicidin in primary monocytes resulted in the loss of TLR2/1-mediated antimicrobial activity against intracellular mycobacteria. Therefore, these data identify a novel mechanism of host defense requiring the induction of IL-1β in synergy with vitamin D activation, for the TLR-induced antimicrobial pathway against an intracellular pathogen.

## Introduction

The innate immune system rapidly responds to infectious pathogens, through recognition of microbial ligands and subsequent triggering of an antimicrobial response. For the intracellular pathogen *Mycobacterium tuberculosis*, a key antimicrobial mechanism involves recognition of bacterial lipoproteins by Toll-like receptors (TLRs), induction of the 25-hydroxyvitamin D3-1α-hydroxylase (CYP27B1), which converts the vitamin D prohormone (25D) into the active 1,25D form, upregulation and activation of the vitamin D receptor (VDR) [1]–[5]. Single nucleotide polymorphisms in the VDR confer increased susceptibility to tuberculosis, suggested a key role for VDR activation in host defense against tuberculosis in humans [6].

It has long been known that activation of the VDR alone in human monocytes induces an antimicrobial activity against *M. tuberculosis*, first demonstrated in the laboratories of Crowle and Rook by addition of the VDR agonist [7], [8]. We reasoned that the VDR-induced antimicrobial pathway involves a set of regulated genes that contribute to host defense against *M. tuberculosis* infection. Vitamin D receptor response elements (VDREs) have been shown to regulate the antimicrobial peptides cathelicidin and defensin beta 4 (DEFB4, formerly HBD2) [1]. Previously, we demonstrated that cathelicidin directly kills *M. tuberculosis* [3], and furthermore, vitamin D-induced expression of cathelicidin was required for antimicrobial activity [4]. In contrast, VDR activation alone was not sufficient to induce DEFB4 expression in monocytes [4]. Here we investigated the mechanism by which activation of the innate system could induce DEFB4 expression in human monocytes.

## Materials and Methods

### Ethics statement

This study was conducted according to the principles expressed in the Declaration of Helsinki. The study was approved by the Institutional Review Board of the University of California at Los Angeles. All donors provided written informed consent for the collection of peripheral blood and subsequent analysis.

### Reagents and bacteria

TLR2/1L is a synthetic 19 kDa *M. tuberculosis* derived lipopeptide (EMC Microcollections, Tuebingen, Germany). Optimal induction of cathelicidin by TLR2/1L was performed at 24 hours based on previous time course experiments (data not shown). 1,25D3 was purchased (BioMol, Plymouth Meeting, PA, USA) and resuspended in sterile filtered ethanol at 10^−2^ M in amber tubes and stored at −80°C in small aliquots. 1,25D3 was added to culture at a concentration of 10^−7^ M to 10^−9^ M, or 10^−8^ M in the absence of a titration, the concentration previously determined to be optimal for provocation of primary human monocytes. VDR antagonist ZK 159 222 (VAZ) was provided from Bayer Schering Pharma AG, and used at 10^−7^ M to 10^−9^ M, or 10^−8^ M in the absence of a titration. *M. tuberculosis* H37Ra strain was a kind gift from Dr. J. Ernst and utilized as described below. Cathelicidin (Phoenix Pharmaceuticals, Burlingame, CA) and DEFB4 (Peptides International, Louisville, KY) peptides were purchased and stored as recommended by the manufacturer. The nonspecific peptide control is the 1–40 amino acids of the amyloid-β protein (Peptides International), selected for its similarity in both length and molecular weight to cathelicidin and DEFB4 peptides, and it has no known antimicrobial activity. Predesigned siRNA oligos were purchased (Dharmacon, Lafayette, CO): siCTRL, siCath, and siDEFB4 were resuspended and stored as recommended by the manufacturer. Cell Line Nucleofection Kit V was purchased and used for siRNA transfections (Amaxa, Gaithersburg, MD). IL-1β neutralizing antibody and recombinant IL-1β were purchased and used at 10 µg/ml and 10 ng/ml respectively (R&D Systems, Minneapolis, MN). NF-κB p65 expression vector was previously described [9].

### Monocyte Isolation and Serum collection

Monocytes were isolated using Ficoll-Paque followed by Percoll gradients (Amersham Biosciences, Piscataway, NJ) and cultured with RPMI containing 10% of vitamin D-sufficient human sera, and stimulated with the indicated conditions. For serum collections, blood was drawn with no anticoagulants, and allowed to clot for one hour. Then the clot was disrupted and centrifuged to separate the serum, which was isolated and filtered. Both 25D and 1,25D levels were measured as previously described [4]. Sera from multiple vitamin D sufficient or borderline insufficient donors were pooled and the bulk sera were supplemented with exogenous 25D3 to achieve 100 nM concentration if needed.

### ELISA and Flow Cytometry

ELISAs were performed as previously described [10]. The IL-1β antibody pair was purchased (Invitrogen, Carlsbad, CA). For measuring IL-1RA levels, we utilized the Human IL-1RA Duoset ELISA kit as recommended by the manufacturer (R&D System). Detection of cell surface IL-1R1 levels was achieved using a three-step flow cytometry assay. Briefly, following TLR2/1L stimulation, cells were harvested from the culture plate and incubated with the primary monoclonal antibody against IL-1R1 (R&D Systems), then a biotinylated secondary antibody, and finally strep-avidin conjugated with a fluorochrome. The three step flow cytometry is necessary for the amplification of the fluorescent signal due to the low expression levels of the IL-1R1 on human primary monocytes.

### Direct antimicrobial activity assay

Our previous studies demonstrate that the attenuated H37Ra strain of *M. tuberculosis* has a similar susceptibility to cathelicidin compared to the virulent H37Rv strain; therefore, we felt that H37Ra is a sufficient model for the effects of antimicrobial peptides on bacterial growth [3], [4]. The direct antimicrobial experiments were performed in conditions that are optimized for cathelicidin activity and *M. tuberculosis* H37Ra grown as previously described [4]. Briefly 2×10^6^ bacteria are incubated with cathelicidin, DEFB4 or a non-specific peptide for two days, and then pulsed with 3 µCi ^3^H-uracil for one day. Following incubation, the bacteria were fixed at a final concentration of 2% paraformaldehyde for 20 minutes then harvested for liquid scintillation counting. Antimicrobial activity was calculated as the percent decrease in CPM values following treatment with the peptide as compared to the no peptide control.

### CFU Assay

Primary human monocytes were infected with *M. tuberculosis* H37Ra at an MOI of 0.5 for 16 hours. The efficiency of infection was determined by auramine rhodamine stain and was regularly in the range of 30%, with an average of 1.1 bacteria per infected cell. Infected cells were washed with 1×PBS to remove extracellular bacteria, counted, and plated into 96 well culture plates. The infected cells were treated with TLR2/1L for three days in triplicate wells, then lysed by resuspension into a 0.3% solution of saponin with vigorous pipetting. Intracellular bacteria were harvested for CFU assay as described [4]. To normalize for the variable starting material per experiment, the relative CFU were calculated by extrapolating the relative values of the TLR2/1L-treated cells to 10^5^ CFU of the media treated cells [3].

### qPCR

RNA was isolated using Trizol Reagent (Invitrogen), and cDNA was synthesized using the iSCRIPT cDNA Synthesis Kit (BioRad, Hercules, CA). The primer sequences for 36B4, DEFB4 and cathelicidin were previously published and methods described [4], [11]. The relative quantities of the gene tested per sample were calculated against the ribosomal protein, large, P0 mRNA (36B4) using the ΔΔC(T) formula as previously described [12].

### Primary monocyte transfection

Transfection of either siRNA or expression constructs into primary human monocytes was accomplished using the Amaxa™ Nucleofection System and the Human Monocyte Kit according to the manufacturer's recommendations. siRNA constructs were used at 100 pmol per transfection, and plasmids at 1 µg per transfection. Transfection efficiency of siRNA into primary monocytes was assessed using siGLO, a fluorescently labeled control oligo (Dharmacon) and averaged 95% transfection rate. Transfection efficiency of expression constructs was assessed using the pMAX-GFP construct (Amaxa), and yielded an average of 38% transfection rate.

## Results

### TLR2/1-activation results in upregulation of DEFB4 mRNA

**Figure 1:**
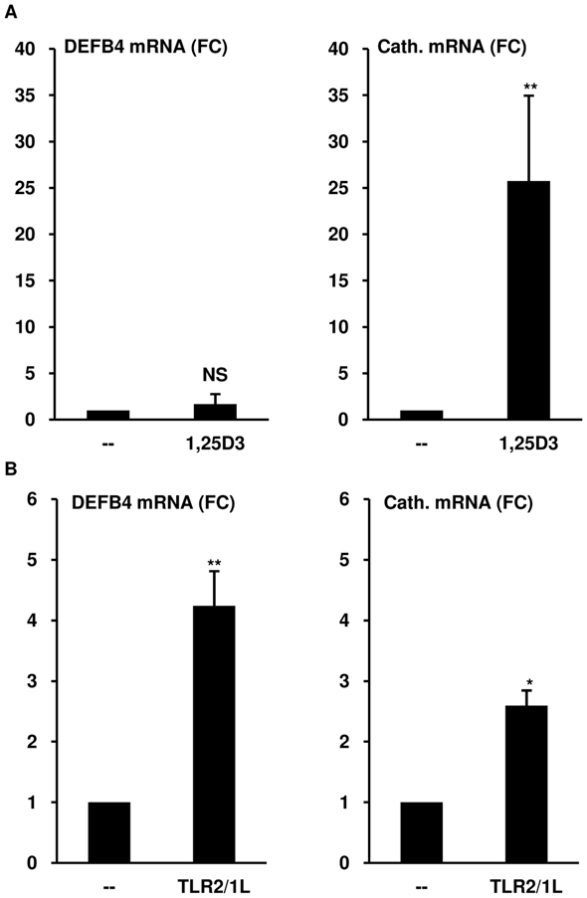
Induction of antimicrobial peptides DEFB4 and cathelicidin from monocytes. (A) Primary human monocytes were stimulated with 1,25D3 at 10^−8^ M for 18 hours. Levels of DEFB4 or cathelicidin mRNA were determined using qPCR. (mean fold change vs media±SEM, n≥5). (B) Monocytes were stimulated with the TLR2/1L (10 µg/ml) for 24 hours. Levels of DEFB4 or cathelicidin mRNA were determined using qPCR. (mean fold change vs media±SEM, n≥4). NS = not significant, * = p≤0.05, ** = p≤0.01.

As previously reported, direct activation of the VDR in primary monocytes with 1,25D3 (10^−8^ M) did not induce DEFB4 mRNA, but was sufficient to induce a 25 fold increase in cathelicidin expression (Student's t-test, p<0.05) (Figure 1a) [4]. Similarly, DEFB4 was not induced by monocytes activated with either 10^−7^ M or 10^−9^ M concentrations of 1,25D3 (data not shown). The induction of cathelicidin by 1,25D3 in human monocytes, and as previously shown the downstream target CYP24A1 suggested that the VDR is functional [4], yet its activation is not sufficient to induce DEFB4.

To determine if other pathways were involved in the induction of DEFB4 in monocytes, we considered TLR2/1 activation, a powerful inducer of innate immune responses in monocytes, including activation of the VDR as well as other signaling pathways. In contrast to 1,25D3 stimulation, activation by the TLR2/1 ligand (TLR2/1L, *M. tuberculosis* derived 19 kDa lipopeptide) resulted in upregulation of DEFB4 mRNA by 4.2 fold (t-test, p<0.005). Cathelicidin was upregulated by 2.6 fold (t-test, p<0.05) in the same experiments (Figure 1b), consistent with previous results [4]. The TLR2/1L-induced DEFB4 mRNA expression levels ranged from two to ten fold over media control, with induction in some donors reaching levels of 30 to 70 fold over media control (data not shown). These data indicate that DEFB4 induction can be triggered in human monocytes through activation of TLR2/1.

### TLR2/1-induced DEFB4 mRNA is dependent on activation of the VDR

**Figure 2:**
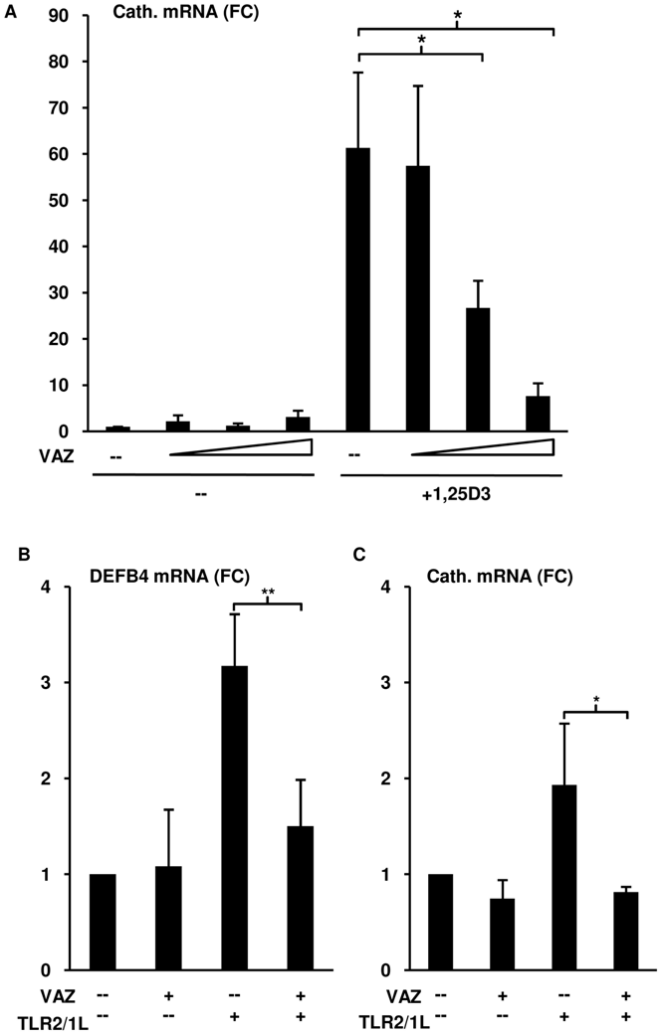
Role of vitamin D receptor activation on the TLR2/1-induced DEFB4 expression. (A) Monocytes were treated with vitamin D receptor antagonist ZK 159 222 (VAZ) at 10^−7^ M, 10^−8^ M, 10^−9^ M or media for 20 minutes then activated with 1,25D3 at 10^−8^ M for 18 hours. Levels cathelicidin mRNA were determined using qPCR. (mean fold change vs media±SEM, n = 4). (B) Monocytes were treated with VAZ 10^−8^ M or media for 20 minutes then activated with the TLR2/1L (10 µg/ml) for 24 hours. Levels of DEFB4 or cathelicidin mRNA were determined using qPCR (mean fold change vs media±SEM, n≥4). * = p≤0.05, ** = p≤0.01.

The ability of TLR2/1L but not 1,25D3 to activate DEFB4 in human monocytes raised the question as to whether VDR activation was required for DEFB4 induction. In order to address this, the VDR antagonist ZK 159 222 (VAZ) was first tested for its ability to block 1,25D3-induced cathelicidin mRNA expression. Preincubation of monocytes with a titration of VAZ (10^−9^ M to 10^−7^ M) reduced 1,25D3-induced (10^−8^ M) cathelicidin mRNA in a dose dependent manner, resulting in a 56% reduction (t-test, p<0.05) at equimolar concentrations and 87% reduction (t-test, p<0.05) at 10-fold molar excess (Figure 2a). Monocytes were then pretreated with VAZ (10^−8^ M) followed by the TLR2/1L for 24 hours. mRNA levels of DEFB4 and cathelicidin were assessed using qPCR. In the presence of VAZ, the TLR2/1L-mediated DEFB4 was reduced by 98% (t-test, p<0.001) and cathelicidin mRNA expression was completely inhibited to levels below unstimulated background calculated as a 120% reduction (t-test, p<0.01) (Figure 2b). Taken together, these data demonstrate that TLR2/1L-mediated induction of DEFB4 mRNA is dependent on activation of the VDR.

### IL-1β is required for TLR2/1L-mediated induction of DEFB4 mRNA

**Figure 3:**
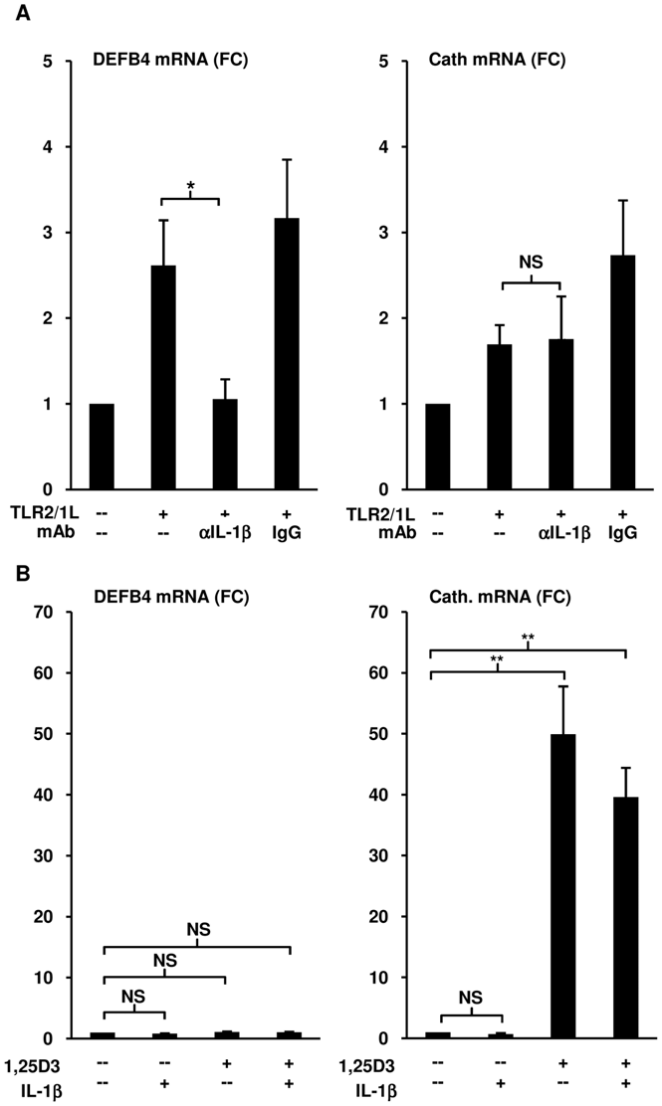
Role of IL-1β activation on DEFB4 expression. (A) Monocytes were incubated with the IL-1β neutralizing antibody (10 µg/ml), isotype control (10 µg/ml) or media for 20 minutes, and then activated with TLR2/1L (10 µg/ml) for 24 hours. Levels of DEFB4 or cathelicidin mRNA were determined using qPCR (mean fold change vs media±SEM, n≥4). (B) Monocytes were stimulated with either media, TLR2/1L (10 µg/ml), recombinant IL-1β (10 ng/ml), or both for 24 hours. Levels of DEFB4 or cathelicidin mRNA were determined using qPCR (mean fold change vs media±SEM, n = 5). NS = not significant, * = p≤0.05, ** = p≤0.01.

The findings that TLR2/1-mediated induction of DEFB4 is VDR-dependent, but 1,25D3 alone was not a sufficient trigger, suggested that in addition, TLR2/1 activation induces a VDR-independent immune mechanism. We hypothesized that IL-1β mediated the TLR2/1-induced DEFB4 expression given that: i) IL-1β was able to trigger DEFB4 expression in epithelial cells [1] and ii) TLR activation of monocytes resulted in the secretion of IL-1β [13], [14]. To determine whether TLR2/1-dependent induction of DEFB4 in primary human monocytes required production of IL-1β, monocytes were pretreated with IL-1β neutralizing monoclonal antibody, or IgG isotype control for 20 minutes then cultured in human serum and stimulated with TLR2/1L. The presence of the IL-1β neutralizing antibody resulted in 97% reduction in the TLR2/1L-induced DEFB4 mRNA expression (t-test, p<0.05), but had no effect on cathelicidin expression (Figure 3a). These data suggest that induction of IL-1β is required for TLR2/1-mediated induction of DEFB4 but not cathelicidin.

Given these results, we tested if co-stimulation of monocytes with IL-1β and 1,25D3 could induce DEFB4 expression. Monocytes stimulated with either media, recombinant human IL-1β (10 ng/ml), 1,25D3 (10^−8^ M), or IL-1β and 1,25D3 together for 24 hours and assayed for DEFB4 and cathelicidin mRNA expression. IL-1β alone did not induce expression of either DEFB4 or cathelicidin mRNA, and 1,25D3 alone induced a 50-fold increase in cathelicidin mRNA expression (t-test, p<0.01) (Figure 3b). However, when added together, IL-1β and 1,25D3 did not induce DEFB4 mRNA, despite a 40-fold upregulation of cathelicidin mRNA (t-test, p<0.01) (Figure 3b). These results indicate that TLR2/1-induced DEFB4 mRNA expression requires the release of both IL-1β and activation of the VDR, but the addition of IL-1β and the VDR agonist together are not sufficient to induce DEFB4 mRNA expression.

### TLR2/1L triggers IL-1β, IL-1R1 and inhibits IL-1RA from monocytes

**Figure 4:**
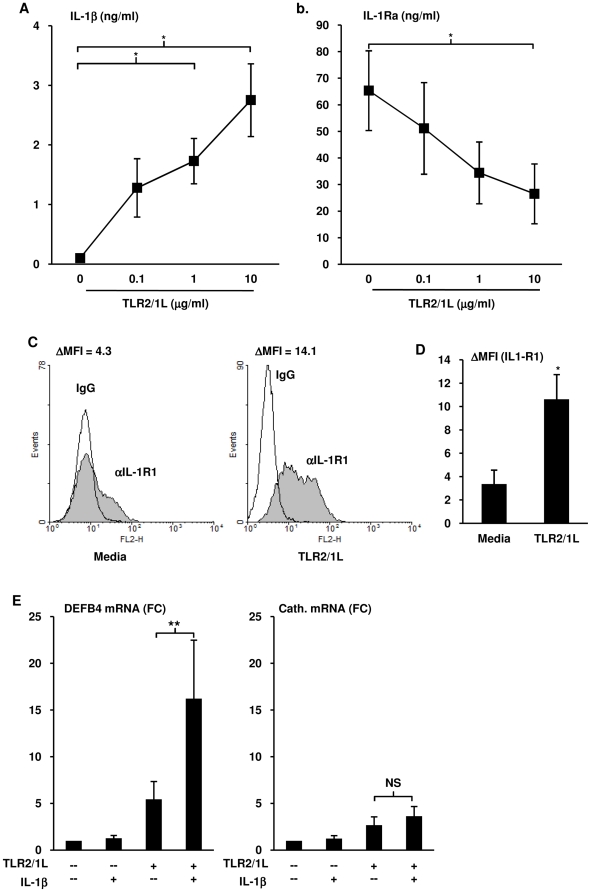
Regulation of IL-1β activity by TLR activation. Monocytes were stimulated with the TLR2/1L at 0.1 µg/ml, 1 µg/ml or 10 µg/ml for 18 hours. (A) IL-1β or (B) IL-1RA levels in culture supernatants were measured using ELISA (mean levels±SEM, n = 5). Monocytes were stimulated with TLR2/1L (10 µg/ml) for 24 hours. Cell surface expression of IL-1R1was assayed using flow cytometry. Data shown are (C) representative of three individual donors, or (D) the mean ΔMFI (n = 3). (E) Monocytes stimulated with either media, TLR2/1L (10 µg/ml), recombinant IL-1β (10 ng/ml) or both for 24 hours. Levels of DEFB4 or cathelicidin mRNA were determined using qPCR (mean fold change vs media±SEM, n = 5). NS = not significant, * = p≤0.05, ** = p≤0.01.

We hypothesized that TLR2/1 activation on monocytes leads to potentiation of IL-1β function based upon the following observations, 1) both IL-1β and activation of the VDR are required for TLR2/1-mediated induction of DEFB4, and 2) co-stimulation with IL-1β and 1,25D3 did not induce DEFB4. Therefore, three key factors that can influence IL-1β activity were assayed in TLR2/1 stimulated monocytes: IL-1β±IL-1 receptor antagonist (IL-1RA) and IL-1 receptor 1 (IL-1R1). Primary human monocytes were isolated and treated with a titration of TLR2/1L (0.1 µg/ml, 1 µg/ml, and 10 µg/ml) for 18 hours, then IL-1β and IL-1RA levels in the culture supernatants were measured using ELISA. TLR2/1 stimulation resulted in significant IL-1β secretion in a dose dependent manner reaching 2.8 ng/ml at the highest TLR2/1L concentration tested (t-test vs media, p<0.05) (Figure 4a). Although monocytes cultured in media alone exhibited spontaneous production of IL-1RA, the addition of TLR2/1L reduced the release of IL-1RA in a dose dependent manner (Figure 4b). At the highest concentration of TLR2/1L (10 ug/ml) IL-1RA was reduced by 60% as compared to media (t-test, p<0.01). Next, cell surface expression of IL-1R1 on monocytes stimulated with either media or TLR2/1L (10 µg/ml) was measured by flow cytometry. TLR2/1 activation resulted in enhanced expression of IL-1R1 (Figure 4c) equaling an average 3.1 fold increase in mean fluorescence intensity (t-test, p<0.05) (Figure 4d). Taken together, these data demonstrate that TLR2/1 stimulation of monocytes increases IL-1β activity through the secretion of IL-1β, increased expression of IL-1R1 and inhibition of IL-1RA.

We hypothesized that TLR2/1 stimulated monocytes would be more sensitive to IL-1β stimulation due to the higher IL-1R1 expression and lower IL-1RA secretion. To test this, primary monocytes cultured in human serum were treated with media, recombinant IL-1β, TLR2/1L, or both simultaneously for 24 hours and assayed for DEFB4 and cathelicidin mRNA expression. Consistent with the previous results, addition of recombinant IL-1β to the culture medium alone did not induce either DEFB4 or cathelicidin mRNA (Figure 4e), and again, TLR2/1L induced expression of DEBF4 and cathelicidin mRNAs. However, when monocytes were co-treated with both IL-1β and TLR2/1L, DEFB4 mRNA expression levels were synergistically increased by three fold over TLR2/1L alone (t-test, p<0.01), but cathelicidin levels were unaffected (Figure 4e). In addition, IL-1β did not increase the TLR2/1-induced expression of either CYP27B1 or the VDR, both key genes in the vitamin D pathway (Figure S1). These experiments demonstrate that TLR2/1 stimulation sensitizes monocytes to IL-1β activation.

### NF-κB activation drives expression of DEFB4 mRNA in monocytes

**Figure 5:**
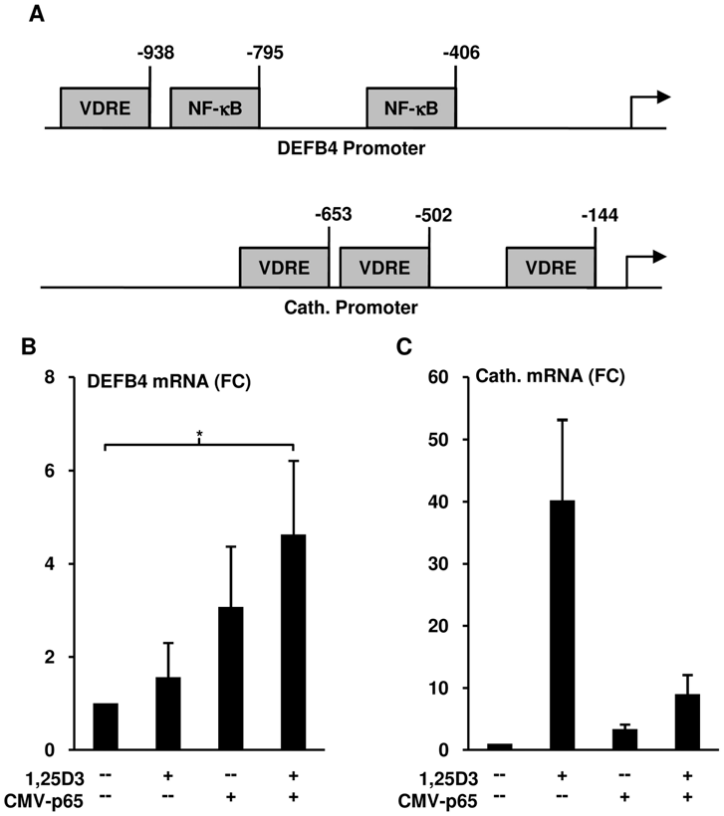
Role of NF-κB activation on TLR2/1-induced DEFB4. (A) Promoter analysis for 1 kb upstream region for cathelicidin and DEFB4, representing NF-κB binding sites and vitamin D response elements (VDRE). Monocytes were transfected with either an NF-κB p65 expression construct (CMV-p65) or a control plasmid, and then stimulated with 1,25D3 (10^−8^ M) for 18 hours. (B) DEFB4 and (C) cathelicidin mRNA levels were determined using qPCR (mean fold change vs media±SEM, n = 6). * = p≤0.05.

In order to gain insight into the underlying mechanism for the differential requirements for IL-1β activation of monocytes in the induction of DEFB4 versus cathelicidin, their respective upstream promoters regions were analyzed for regulatory elements by MatInspector. Three vitamin D receptor response elements (VDREs) were located in the cathelicidin promoter at positions −144, −502, and −653 as compared to only one in the DEFB4 promoter at position −938 (Figure 5a). Interestingly, there were two NF-κB response elements found in the DEFB4 promoter at positions −406 and −795, whereas none were present in the cathelicidin promoter region (Figure 5a). Previously published studies have verified all of the above mentioned response elements to be *bona fide* promoter elements for their respective transcription factors [1], [15].

Based upon the presence of two NF-κB response elements in the DEFB4 promoter, we determined whether NF-κB activation could trigger monocytic expression of DEFB4 mRNA. IL-1β has been shown to induce the activation and nuclear translocation of the p65 and p50 NF-κB subunits, but does not activate c-Rel, RelB or p52 in epithelial cells [15], [16]. Therefore, primary human monocytes were transfected with a plasmid expressing the p65 NF-κB subunit under control of the CMV-promoter, or a CMV-GFP control plasmid. In the context of CMV-p65 transfection, 1,25D3 stimulation of monocytes resulted in a significant 4.6 fold increase in DEFB4 mRNA expression (t-test, p<0.05, n = 6), whereas in the CMV-GFP control transfected cells, 1,25D3 did not induce DEFB4 expression (Figure 5b). CMV-p65 also induced a trend for increasing DEFB4 expression (t-test, p = 0.14, n = 6) in the absence of additional 1,25D3 (Figure 5b). On the other hand, transfection of CMV-p65 trended towards inhibition of the 1,25D3 induced cathelicidin expression by 77% (t-test, p = 0.08, n = 6) as compared to the 1,25D3 elicited cathelicidin expression in the CMV-GFP control transfected cells (Figure 5b). These data suggest that in human monocytes, activation of both NF-κB and VDR response elements are required for optimal induction of DEBF4.

### DEFB4 peptide exhibits direct antimicrobial activity

**Figure 6:**
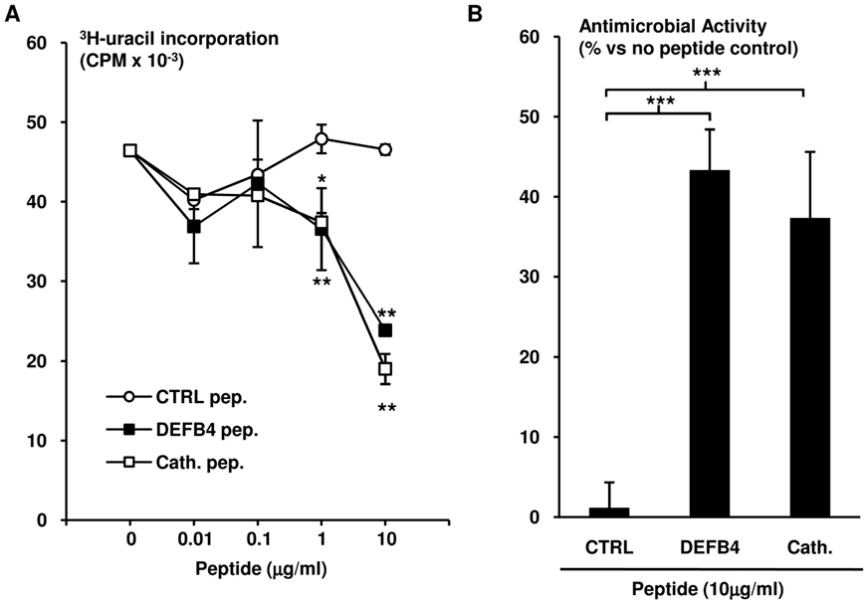
Direct antimicrobial activity of DEFB4 and cathelicidin peptides. *M. tuberculosis* H37Ra were incubated with a control peptide, DEFB4 peptide or cathelicidin peptide at 0.01 µg/ml, 0.1 µg/ml, 1 µg/ml and 10 µg/ml as well as media for three days. Bacterial proliferation was assayed using ^3^H-uracil up take. Data shown is (A) representative of three independent experiments, and (B) mean antimicrobial activity±SEM (n≥5). * = p≤0.05, ** = p≤0.01, *** = p≤0.001.

Although human DEFB4 has been demonstrated to be associated with *M. tuberculosis* in alveolar macrophages during infection [17], the antimicrobial activity of DEFB4 against the bacterium has not been studied. To address this, *M. tuberculosis* (attenuated H37Ra strain) were incubated with DEFB4 peptide, or cathelicidin peptide (LL-37), as well as an unrelated control peptide (CTRL), at a range from 0.01 µg/ml to 10 µg/ml for three days. Bacterial viability was then assessed using ^3^H-uracil uptake assay. Cathelicidin peptide demonstrated a dose dependent antimicrobial activity, corroborating previous results [2], [4]. The DEFB4 peptide demonstrated similar dose dependent antimicrobial capacity to cathelicidin. In contrast, the control peptide was unable to reduce bacterial viability (Figure 6a). The observed antimicrobial activity for DEFB4 and cathelicidin at 10 µg/ml compared to the control peptide was calculated as 44% (t-test, p<0.001) and 41% (t-test, p<0.001), respectively (Figure 6b). These results demonstrate that DEFB4 is directly antimicrobial against *M. tuberculosis*.

### Both DEFB4 and cathelicidin are required for TLR2/1-mediated antimicrobial activity

We have previously demonstrated that induction of cathelicidin was required for 1,25D3-mediated antimicrobial activity [3]. To compare the role of DEFB4 and cathelicidin in the TLR2/1-mediated antimicrobial pathway, siRNA technology was used to knock down specific gene expression. Primary human monocytes were transfected with siRNA oligos complementary to the DEFB4 mRNA (siDEFB4), the cathelicidin mRNA (siCath), or a non-specific control oligo (siCTRL). The transfected cells were treated with the TLR2/1L for 24 hours, and then expression of cathelicidin and DEFB4 mRNAs were measured. siDEFB4 and siCath both completely knocked-down the TLR2/1L-induced expression of their respective targets, whereas siCTRL had no effect (Figure S2).

**Figure 7:**
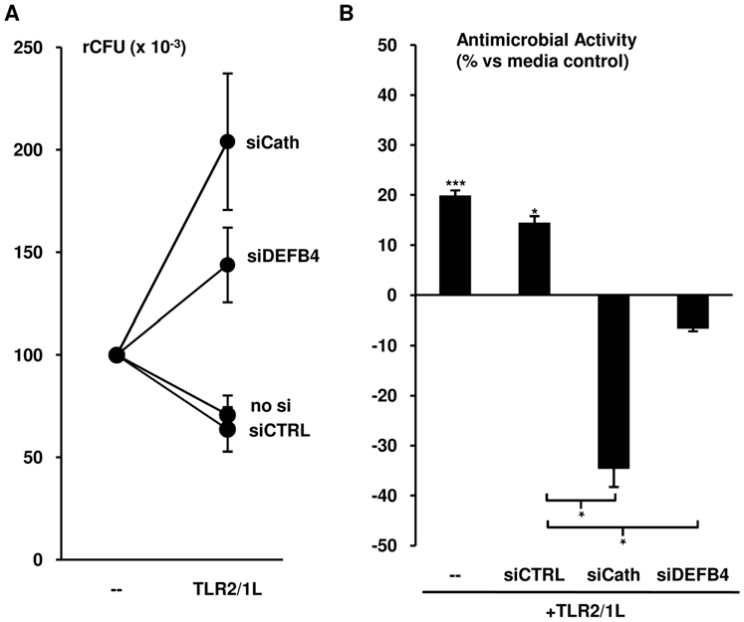
Role of DEFB4 and cathelicidin in TLR-mediated antimicrobial activity. Monocytes were transfected with either siRNA oligos specific for DEFB4 (siDEFB4) or cathelicidin (siCath) as well as a non-specific siRNA oligo (siCTRL) or no siRNA. The cells were then infected with *M. tuberculosis* H37Ra at an MOI of 0.5 for 18 hours followed by treatment with the TLR2/1L for three days. Intracellular bacterial load was measured using CFU assay. Data shown are (A) representative of six individual experiments, and (B) mean antimicrobial activity±SEM (n≥6). * = p≤0.05, *** = p≤0.001.

Subsequently, monocytes transfected with siDEFB4, siCath and siCTRL as well as untransfected monocytes were infected with *M. tuberculosis* H37Ra at an MOI of 0.5 for 18 hours. The infected cells were then treated with media or TLR2/1L for three days, and their intracellular bacterial viability was determined by colony forming units assay (CFU). In one representative experiment, treatment with TLR2/1L reduced the viability of the intracellular bacteria in the untransfected and siCTRL transfected cells by 30% and 36% respectively (Figure 7a). In contrast, transfection of monocytes with siCath or siDEFB4 ablated the TLR2/1L-induced antimicrobial activity (Figure 7a). TLR2/1L treatment of untransfected and siCTRL transfected monocytes resulted in an average antimicrobial activity of 20% (t-test vs media, p<0.001) and 14% (t-test vs media, p<0.05), respectively (Figure 5b). However, transfection with siCath or siDEFB4 resulted in a 134% and 107% reduction of TLR2/1L-induced antimicrobial activity as compared to siCTRL transfected cells, respectively (t-test, p<0.05), indicating enhancement of bacterial growth (Figure 7b). These data suggest that the TLR2/1L-induced expression of both DEFB4 and cathelicidin are required for optimal antimicrobial activity against intracellular mycobacterial infection in human monocytes.

## Discussion

**Figure 8:**
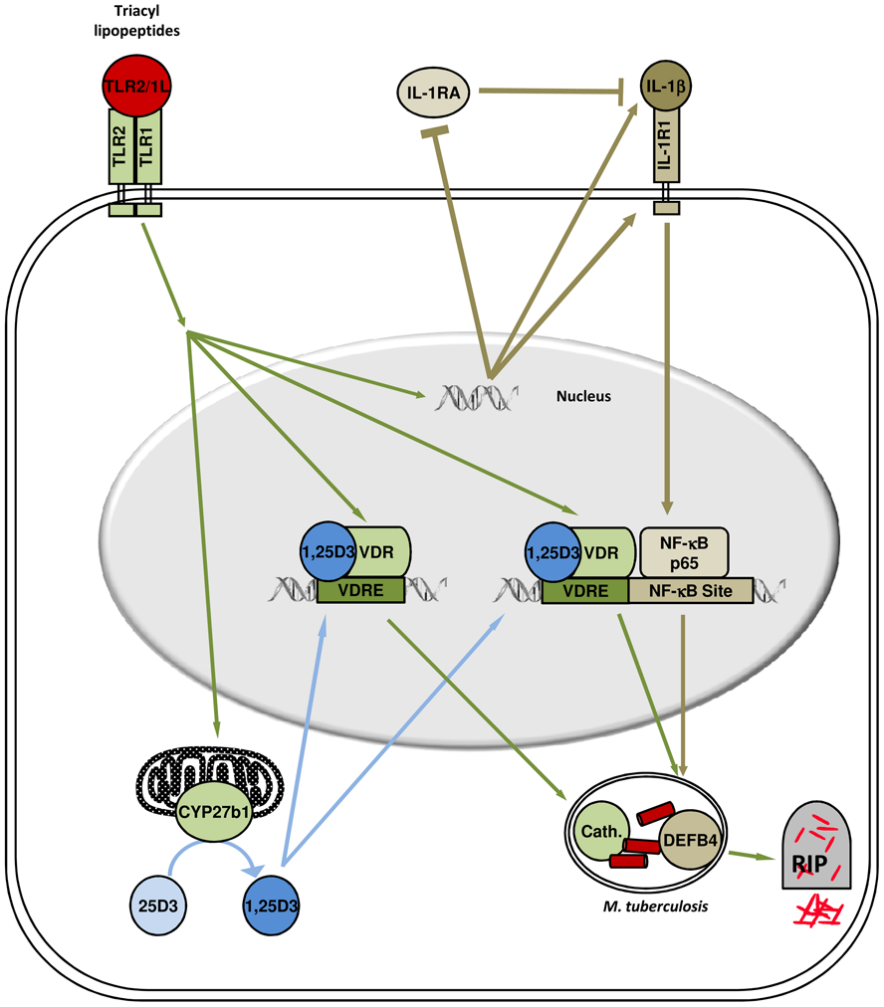
Model. Human TLR2/1L-mediated antimicrobial activity in monocytes requires two distinct pathways. Triggering TLR2/1L results in activation of the vitamin D pathway, including expression of the VDR and CYP27B1, leading to induction of cathelicidin. On the other hand, TLR-mediated expression of DEFB4 requires convergence of the IL-1β and VDR pathways. Activation of TLR2/1 results in regulation of IL-1β activity, through the release of IL-1β expression of its receptor, IL-1R1, and simultaneous downregulation of the IL-1RA. Both cathelicidin and DEFB4 are required for TLR2/1-mediated antimicrobial activity.

The mechanisms by which TLR2/1 activation of monocytes triggers a vitamin D-dependent induction of antimicrobial activity are central to host defense against intracellular *M. tuberculosis* [4]. Here we demonstrate that convergence of IL-1β and vitamin D transcriptional activation induced expression of the antimicrobial peptide DEFB4. Importantly, TLR2/1L activation triggered IL-1β activity, involving the upregulation of both IL-1β and IL-1 receptor, and downregulation of the IL-1 receptor antagonist (Figure 8). TLR2/1L induction of IL-1β was required for upregulation of DEFB4, but not cathelicidin, whereas VDR activation was required for expression of both antimicrobial genes. A synergy between NF-κB and VDR activation was sufficient to upregulate expression of DEFB4. Finally, knockdown of either DEFB4 or cathelicidin resulted in the loss of TLR2/1-induced antimicrobial activity against intracellular mycobacteria. Therefore, these data identify a novel mechanism of host defense requiring the induction of two distinct signaling pathways, involving IL-1βin synergy with vitamin D activation, for TLR-induced antimicrobial activity against an intracellular pathogen.

Upon triggering TLR2/1 with lipopeptide, both human and murine macrophages activate antimicrobial pathways against intracellular mycobacteria [4], [18]. In comparing the human and mouse TLR-induced antimicrobial pathways, it is noteworthy that the human antimicrobial response requires induction of multiple antimicrobial peptides, both cathelicidin and DEFB4, as opposed to the murine antimicrobial response which is dependent upon generation of nitric oxide [18]–[20]. It remains to be determined if the cathelicidin- and DEFB4-mediated antimicrobial activity is bactericidal or bacteriostatic. In humans, both the cathelicidin and DEFB4 promoters contain VDREs [1], VDR activation was required for their induction, as well as TLR-induced antimicrobial activity [4]. In contrast, there are no VDRE sites present in the mouse cathelicidin promoter region [21], and to our knowledge there are no other known vitamin D-dependent antimicrobial peptides in the murine genome. Interestingly, 1,25D induces nitric oxide generation in murine macrophages [22], although the role for vitamin D in this murine TLR-induced antimicrobial response remains to be determined. Although our data indicate that the human innate immune system has evolved a powerful antimicrobial mechanism against intracellular mycobacterial infection through the vitamin D dependent production of antimicrobial peptides, it does not preclude a role for nitric oxide [6], [23], [24] and other mechanisms such as superoxide generation and autophagy [6], [25].

Although VDR activation was required for induction of both DEFB4 and cathelicidin, neutralization of IL-1β prior to TLR-activation resulted in the loss of DEFB4 but not cathelicidin. Analysis of the cathelicidin and DEFB4 promoter regions revealed two NF-κB response elements present in the DEFB4 promoter region, and none in the cathelicidin promoter. In contrast, both genes contained vitamin D response elements, one in DEFB4 promoter and three in cathelicidin. These data suggested a potential mechanism by which IL-1β could uniquely influence the TLR-induced expression of DEFB4 and not cathelicidin while both genes shared a requirement for vitamin D. Transfection of NF-κB p65, the predominant IL-1β induced NF-κB subunit [15], [16], into monocytes resulted in a significant expression of DEFB4 only with VDR activation. In contrast, NF-κB p65 transfection decreased 1,25D3 induced cathelicidin, further highlighting the differential regulation of these two antimicrobial genes. However, the mechanism by which NF-κB p65 transfection inhibits cathelicidin is currently unclear and warrants further exploration. Taken together these data demonstrate a novel role for IL-1β in the antimicrobial activity against intracellular pathogens, through the induction of DEFB4 in synergy with vitamin D. Furthermore, this suggests that there are two distinct vitamin D-dependent TLR-induced antimicrobial mechanisms, with DEFB4 expression also dependent on induction of IL-1β.

In addition to induction of IL-1β expression and VDR activation, triggering of TLR2/1 was found to modulate IL-1β activity by increasing the cell's responsiveness to the secreted IL-1β. The monocyte response to IL-1β was upregulated by TLR2/1 activation through the simultaneous secretion of IL-1β, upregulation of cell surface IL-1R1 and downregulation of baseline IL-1RA. Addition of IL-1β to TLR2/1L stimulated monocytes resulted in synergistic upregulation of DEFB4, which reflects the increased IL-1β responsiveness. On the other hand, IL-1β plus 1,25D3 did not induce expression of DEFB4, presumably because the IL-1R1 was not upregulated and/or the IL-1RA was not downregulated. These findings provide a potential molecular mechanism for the previously known association of a high IL-1β/low IL-1RA producing haplotype with pleural tuberculosis, a form of the disease that generally resolves without chemotherapy [26]. In addition, there is also an elevated level of 1,25D in the pleural effusion fluid compared to serum of the same individual, resulting in an optimal local microenvironment for the induction of antimicrobial responses, including cathelicidin and DEFB4 [27]. Furthermore, IL-1R1 has been demonstrated to be required for host defense against *M. tuberculosis* in a murine model [28]. Given that excessive activation of IL-1β results in tissue injury and sepsis [29], IL-1β activity is subject to negative feedback by the IL-1RA [30]. While both IL-1β and IL-1RA are produced during inflammatory conditions, IL-1RA is also induced by anti-inflammatory signals [29], [31]–[36]. As such, the balance between IL-1β and IL-1RA during an innate immune response plays a major role the pathogenesis of inflammatory diseases such as diabetes, arthritis, inflammatory bowel disease [37], and as presented here, TLR-mediated host defense mechanisms.

Although TLR activation induces DEFB4 in both monocytes and epithelial cells [4], [38], [39], IL-1β alone was not sufficient to induce expression of DEFB4 in monocytes, yet it is a potent inducer of DEFB4 in epithelial cells [40]–[42]. The differential regulation of antimicrobial peptide expression in monocytes vs. epithelial cells reflects the location of these cell types and the immunomodulatory properties of antimicrobial peptides. Epithelial cells form the barrier at which pathogens are initially encountered, and they efficiently utilize the antimicrobial peptide family of genes, for host defense at body surfaces interacting with the outside environment [38], [43]–[45]. However, in addition to their antimicrobial activity, β-defensins and cathelicidin trigger inflammation, by their chemotactic activity for innate and adaptive immune cells such as monocytes, neutrophils, mast cells, dendritic cells and T cells [46]. At body surfaces the antimicrobial peptides are rapidly shed, such that this inflammatory activity is transient, but when released in tissues, the resulting inflammatory cell infiltrate contributes to immune-mediated injury, a complication of host defense in chronic infectious diseases including mycobacterial infections such as leprosy, tuberculosis and Buruli ulcer. Therefore, the more stringent regulation for monocytic expression of cathelicidin and DEFB4 could potentially represent a mechanism to prevent tissue injury in confined spaces.

IL-1β is clearly pivotal to host defense against microbial pathogens, required for cell recruitment and induction of inflammation, but also augmenting acquired lymphocyte responses [29], [47]. In murine models, a key role for IL-1 has been shown in immunity to a variety of pathogens, including *Listeria monocytogenes*, *Staphylococcus aureus*, and *Helicobacter pylori* [47]–[51]. Here, we have identified a novel mechanism by which IL-1β contributes to human host defense, requiring convergence of the TLR-induced IL-1β and VDR pathways, to trigger expression DEFB4. Furthermore, the data establish that the antimicrobial peptides, DEFB4 and cathelicidin, are required effector molecules in the TLR-induced antimicrobial response against intracellular mycobacteria in macrophages. Elucidation of the immune defense mechanisms utilized by human macrophages to combat pathogens provides possible targets for the development of new therapeutic strategies, potentially useful given the emergence of multidrug resistant strains of *M. tuberculosis* and other lethal microbes.

## Supporting Information

Figure S1 (0.14 MB PDF)

Figure S2 (0.12 MB PDF)
