# Supplementary material for: Convergence of IL-1β and VDR Activation Pathways in Human TLR2/1-Induced Antimicrobial Responses
Source: PLoS One. 2009 Jun 5;4(6):e5810. doi: 10.1371/journal.pone.0005810 (PMC2686169; doi:10.1371/journal.pone.0005810)
Supplement: Figure S1 — (0.14 MB PDF) [file pone.0005810.s001.pdf]

Figure S1

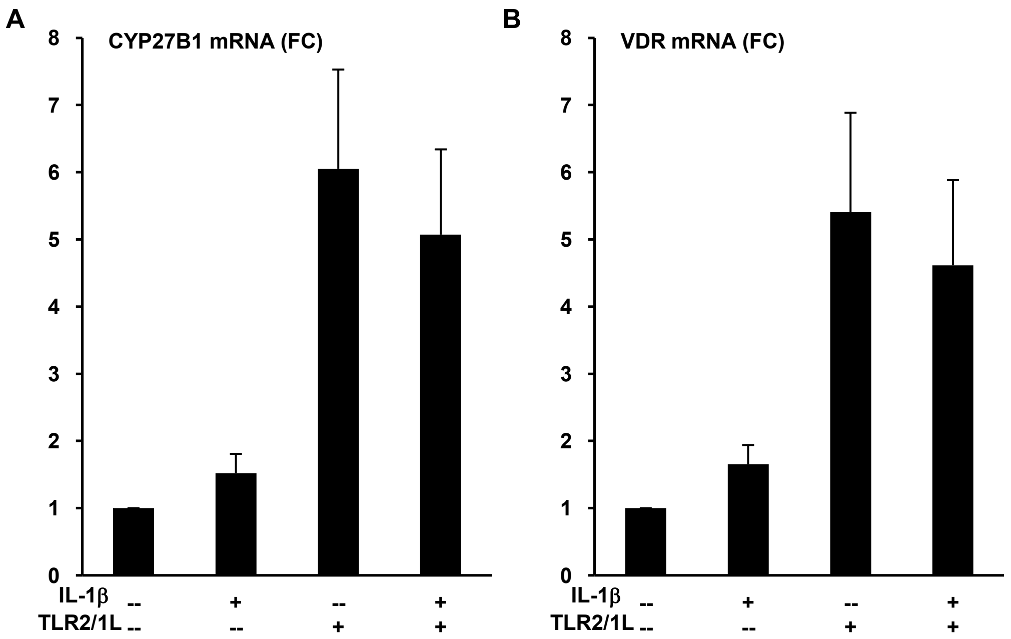

Figure S1. Monocytes stimulated with either media, TLR2/1L (10 μg/ml), recombinant IL-1β (10 ng/ml) or both for 24 hours. Levels of (A) CYP27B1 or (B) VDR mRNA were determined using qPCR (mean fold change vs media ± SEM, n = 5).
