# Supplementary material for: Convergence of IL-1β and VDR Activation Pathways in Human TLR2/1-Induced Antimicrobial Responses
Source: PLoS One. 2009 Jun 5;4(6):e5810. doi: 10.1371/journal.pone.0005810 (PMC2686169; doi:10.1371/journal.pone.0005810)
Supplement: Figure S2 — (0.12 MB PDF) [file pone.0005810.s002.pdf]

Figure S2

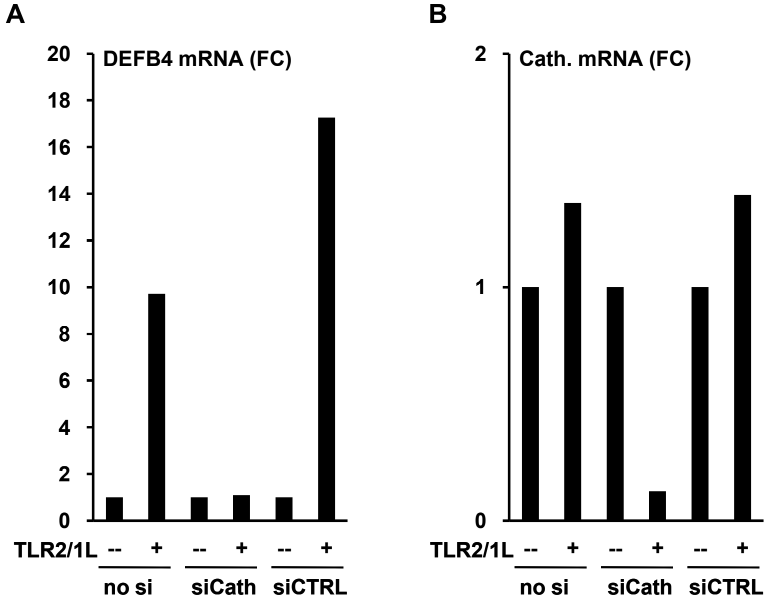

Figure S2. Monocytes were transfected with either siRNA oligos specific for DEFB4 (siDEFB4) or cathelicidin (siCath) as well as a non-specific siRNA oligo (siCTRL) or no siRNA. The cells were then stimulated with the TLR2/1L for three days. Levels of (A) DEFB4 or (B) cathelicidin mRNA were determined using qPCR . Data shown is representative of three separate experiments.
